# Hematological Changes in Pulmonary Tuberculosis: Focus on Anemia, Disease Severity, and Therapeutic Implications

**DOI:** 10.7759/cureus.86550

**Published:** 2025-06-22

**Authors:** Devendra N Tiu, Siddiqui Mahaiboob Fatima M Sirajuddin Ahmed Siddiqi, Rajesh Ranjan

**Affiliations:** 1 Physiology, Sheikh Bhikhari Medical College, Hazaribagh, IND; 2 Physiology, Dr. Balasaheb Vikhe Patil Rural Medical College, Loni, IND; 3 Epidemiology, National Institute of Health and Family Welfare, New Delhi, IND

**Keywords:** anemia, cross-sectional study, disease severity, hematological parameters, pulmonary tuberculosis

## Abstract

Background

Anemia is a common complication in patients with pulmonary tuberculosis (TB), potentially affecting treatment outcomes and overall health. This study aimed to evaluate the hematological changes associated with pulmonary TB, focusing on the severity of anemia and its correlation with disease severity.

Methods

This cross-sectional study was conducted at Rajendra Institute of Medical Sciences (RIMS), Ranchi, involving 76 patients diagnosed with pulmonary TB and a matched control group of 76 healthy individuals. Hematological parameters, including hemoglobin levels, hematocrit, and anemia classification, were assessed at admission and during follow-up at two and six months. Correlations between anemia severity and TB severity were analyzed using Pearson’s correlation coefficient.

Results

The mean age of participants in the pulmonary TB group was 36.2 ± 15.6 years. A significant prevalence of anemia was observed in the pulmonary TB group, affecting 67 out of 76 participants (88%), compared to 12 out of 76 participants (15.8%) in the control group, with a p-value of <0.0001. Normocytic anemia was the most common type, affecting 45 out of 76 TB patients (60%). Hemoglobin levels were significantly lower in the TB group (10.10 ± 1.73 g/dL) compared to controls (13.07 ± 1.14 g/dL), with a p-value of <0.0001. Correlation analysis showed a strong negative correlation between hemoglobin levels and TB severity (r = -0.58, p < 0.0001) and duration of symptoms (r = -0.49, p < 0.0001).

Conclusion

Anemia is prevalent among patients with pulmonary TB and is significantly associated with disease severity and duration of symptoms. These findings underscore the importance of regular hematological monitoring and management of anemia in TB patients to improve clinical outcomes.

## Introduction

Tuberculosis (TB), caused by *Mycobacterium tuberculosis*, remains one of the leading infectious causes of morbidity and mortality globally, particularly in low- and middle-income countries like India [1]. According to the World Health Organization (WHO), in 2022, an estimated 10.6 million new TB cases were reported worldwide, with India alone accounting for over a quarter of the global burden [2]. Pulmonary TB, which affects the lungs, is the most prevalent form of the disease and is often accompanied by systemic manifestations, including a range of hematological abnormalities that can significantly impact patient outcomes [3].

Hematological changes in TB patients are well-documented, with anemia being among the most common findings, affecting 30-85% of individuals with active disease [4]. The pathophysiology of anemia in TB is complex and multifactorial, driven by chronic inflammation, impaired iron metabolism, and the effects of malnutrition and micronutrient deficiencies [5]. Anemia of chronic disease (ACD) is frequently observed in these patients, characterized by hypoferremia, normal or increased ferritin levels, and reduced erythropoiesis [5]. Additionally, iron deficiency anemia (IDA) can coexist or overlap with ACD, particularly in populations with a high prevalence of malnutrition, such as those in India [6].

The impact of anemia in TB extends beyond its effects on general well-being; it is also closely associated with the clinical course of the disease [7]. Anemia has been linked to increased disease severity, higher bacterial loads, and delayed sputum conversion, all of which contribute to prolonged infectious periods and worse clinical outcomes [8]. Studies have shown that the severity of anemia in TB patients can correlate with markers of disease activity, such as elevated inflammatory cytokines (e.g., interleukin 6 (IL-6) and tumor necrosis factor alpha (TNF-α)) and C-reactive protein (CRP) levels [7,8]. These inflammatory mediators disrupt iron homeostasis by upregulating hepcidin, leading to reduced iron availability for erythropoiesis, thereby perpetuating anemia [8].

The interplay between TB and anemia also raises important considerations for therapeutic management. While standard antituberculosis therapy (ATT) remains the cornerstone of TB treatment, the resolution of anemia during ATT can be variable [9]. For patients with significant iron deficiency or severe anemia, adjunctive therapies, such as iron supplementation or erythropoiesis-stimulating agents, may be necessary to improve outcomes [10]. However, indiscriminate iron supplementation can pose risks, including the potential for increased bacterial growth in iron-rich environments [10]. Thus, understanding the specific types and severity of anemia in TB patients is critical for optimizing therapeutic strategies [9].

Despite the recognized association between TB and hematological changes, there remains a gap in research regarding the comparative analysis of these changes in TB patients versus healthy controls, particularly in resource-limited settings [10]. Additionally, few studies have thoroughly explored the prognostic value of these hematological parameters in assessing disease severity and predicting treatment response [11-14]. This study aimed to evaluate the spectrum of hematological changes, especially focusing on anemia, in patients with pulmonary TB. It further seeks to assess the correlation between these changes and the severity of TB, explore their prognostic significance, and determine the potential need for supplementary therapies, such as iron supplementation, alongside conventional ATT. This investigation could provide critical insights into the comprehensive management of TB, thereby improving patient outcomes in settings with high disease burden.

## Materials and methods

Study design and setting

This cross-sectional study was conducted in the Physiology Department at the Rajendra Institute of Medical Sciences (RIMS) in Ranchi. The department was responsible for laboratory investigations, while participant recruitment was carried out in collaboration with the Outpatient Departments (OPDs) and inpatient wards of the Medicine and TB and Chest Departments. The study was conducted over a two-year period from April 2021 to May 2023 and involved both pulmonary TB patients and healthy controls for comparative analysis.

Study population

The study comprised two groups: pulmonary TB patients and age- and sex-matched healthy controls. The pulmonary TB group included patients aged 18 years and above diagnosed with active pulmonary TB based on clinical evaluation and at least one of the following criteria: positive sputum smear microscopy for acid-fast bacilli (AFB), GeneXpert confirmation of *Mycobacterium tuberculosis *with or without rifampicin resistance, or positive mycobacterial culture. To ensure homogeneity in pulmonary disease assessment, patients with disseminated TB were excluded. Participants were recruited from individuals attending the RIMS OPD with symptoms such as cough and hemoptysis or from untreated sputum-positive cases admitted to the Medicine and TB and Chest wards. The control group included 76 healthy individuals aged 20-70 years with no history of TB or chronic illnesses. These controls were selected from medical students, family members, interns, postgraduate medical students, nurses, security guards, and paramedical staff at RIMS. A detailed history and clinical examination were conducted to exclude any major diseases. Individuals with conditions that could confound hematological analysis were excluded. These conditions included bleeding disorders (e.g., hemorrhoids, melena, peptic ulcer disease, menorrhagia, hematuria), malignancies, HIV infection, autoimmune diseases, and chronic liver or kidney diseases.

Sample size

The sample size was calculated based on the expected prevalence of anemia in pulmonary TB patients. Using the formula for comparing means between two groups, a clinically significant difference of 3 g/dL in mean hemoglobin levels between TB patients and healthy controls was considered, with a pooled standard deviation of approximately 1.35 g/dL. A minimum of 76 participants per group was required to achieve 80% power at a 5% significance level.

Data collection

Data collection involved recording demographic details, clinical history, and laboratory findings using a structured proforma. Information included age, sex, duration of symptoms, and history of previous TB treatment. The severity of TB was classified based on clinical parameters and chest X-ray findings in accordance with the National Tuberculosis Elimination Programme (NTEP) guidelines.

Laboratory investigations

Blood samples (5 mL) were collected via venipuncture into ethylenediaminetetraacetic acid (EDTA) tubes and processed for hematological analysis within two hours of collection. Complete blood count (CBC) parameters included hemoglobin concentration, hematocrit, red blood cell (RBC) indices (mean corpuscular volume (MCV), mean corpuscular hemoglobin (MCH), and mean corpuscular hemoglobin concentration (MCHC)), total and differential white blood cell (WBC) count, and platelet count. Peripheral blood smear analysis was performed to assess RBC morphology and classify anemia types. Serum iron studies, including serum ferritin, serum iron, total iron-binding capacity (TIBC), and transferrin saturation, were conducted to differentiate iron deficiency anemia (IDA) from anemia of chronic disease (ACD). Inflammatory markers, such as CRP and erythrocyte sedimentation rate (ESR), were measured to evaluate systemic inflammation. Hematology analyses were performed using an automated analyzer (Mindray BC-6800, Mindray Medical International Limited, Shenzhen, China) with daily calibration and proficiency testing to ensure accuracy. Serum iron studies were conducted using immunoassays, with internal controls run alongside patient samples for reliability.

Assessment of anemia and disease severity

Anemia was defined as hemoglobin levels below 13 g/dL for males and below 12 g/dL for females, following the WHO criteria. Based on MCV values, anemia was further classified into microcytic, normocytic, and macrocytic types. The severity of anemia was categorized as mild, moderate, or severe according to WHO guidelines. Disease severity was assessed through chest X-ray findings, classified as minimal, moderate, or advanced disease based on NTEP guidelines. The extent of lung involvement was graded based on the number of lung zones affected: minimal (1-2 zones), moderate (3-4 zones), and advanced (>4 zones). Additional parameters, such as body mass index (BMI), symptom duration, and the presence of systemic symptoms (e.g., fever, night sweats, and weight loss), were also evaluated to determine disease severity.

Statistical analysis

Data were entered into MS Excel (Microsoft Corporation, Redmond, Washington, United States) and analyzed using IBM SPSS Statistics for Windows, Version 26 (Released 2019; IBM Corp., Armonk, New York, United States). Continuous variables were expressed as mean ± standard deviation (SD), while categorical variables were presented as frequencies and percentages. Comparisons between the TB and control groups were made using the independent t-test for continuous variables and the chi-square test for categorical variables. The relationship between anemia severity and TB severity was evaluated using Pearson’s or Spearman’s correlation coefficient, as appropriate. Multivariate logistic regression analysis was performed to determine predictors of severe anemia in TB patients. A p-value of <0.05 was considered statistically significant.

Ethical considerations

The study protocol was approved by the Institutional Ethics Committee (approval number: IEC/SBMC/2021/343). A written informed consent was obtained from all participants before enrollment, ensuring confidentiality and voluntary participation. All study procedures adhered to the ethical principles outlined in the Declaration of Helsinki.

## Results

The study included 76 participants in both the pulmonary TB and control groups. The mean age of participants in the pulmonary TB group was 36.2 ± 15.6 years, while in the control group, it was 37.8 ± 14.7 years, with no significant difference (p = 0.514). Gender distribution was similar, with 55 (72.4%) males and 21 (27.6%) females in the pulmonary TB group, compared to 53 (69.7%) males and 23 (30.3%) females in the control group (p = 0.720). The mean duration of symptoms before diagnosis in the pulmonary TB group was 7.5 ± 2.3 weeks, and previous TB treatment was reported in 10 (13.2%) participants. The mean BMI was significantly lower in the pulmonary TB group (18.9 ± 2.4 kg/m²) compared to the control group (22.1 ± 2.6 kg/m², p < 0.0001) (Table 1).

**Table 1:** Demographic and clinical characteristics of participants with pulmonary tuberculosis and healthy controls TB: tuberculosis

Characteristic	Pulmonary TB group (n = 76)	Control group (n = 76)	p-value	t-value/Chi-square value/F-value
	Frequency (%)/mean ± SD			
Age (years)	36.2 ± 15.6	37.8 ± 14.7	0.514	t = -0.654
Gender				
Male	55 (72.4%)	53 (69.7%)	0.720	χ² = 0.129
Female	21 (27.6%)	23 (30.3%)		
Duration of symptoms (weeks)	7.5 ± 2.3	-	-	-
Previous TB treatment	10 (13.2%)	-	-	-
BMI (kg/m²)	18.9 ± 2.4	22.1 ± 2.6	<0.0001	t = -8.015

In the pulmonary TB group, hemoglobin levels significantly increased from 10.10 ± 1.73 g/dL at admission to 12.11 ± 1.57 g/dL at two months and 13.40 ± 0.61 g/dL at six months (control: 13.07 ± 1.14 g/dL, p < 0.0001). Hematocrit levels also improved from 31.4 ± 5.2% at admission to 34.7 ± 4.8% at two months and 38.2 ± 2.9% at six months (control: 38.2 ± 4.5%, p < 0.0001). RBC counts rose from 4.2 ± 0.7 × 10⁵ cells/µL at admission to 4.5 ± 0.5 × 10⁵ cells/µL at two months and 4.7 ± 0.4 × 10⁵ cells/µL at six months (control: 4.9 ± 0.6 × 10⁵ cells/µL, p < 0.0001). Platelet counts decreased over time, from 315.2 ± 55.3 × 10³ cells/µL at admission to 275.3 ± 46.4 × 10³ cells/µL at six months (control: 276.7 ± 52.3 × 10³ cells/µL, p < 0.0001) (Table 2).

**Table 2:** Hematological parameters at admission and follow-up in pulmonary tuberculosis patients and healthy controls TB: tuberculosis; RBC: red blood cell; MCV: mean corpuscular volume; MCH: mean corpuscular hemoglobin; MCHC: mean corpuscular hemoglobin concentration

Parameter	Pulmonary TB group (n = 76) mean ± SD			Control group (n = 76) mean ± SD	p-value	t-value/F-value
	At admission (n = 76)	At 2 months (n = 55)	At 6 months (n = 44)			
Hemoglobin (g/dL)	10.10 ± 1.73	12.11 ± 1.57	13.40 ± 0.61	13.07 ± 1.14	<0.0001	t = -12.153
Hematocrit (%)	31.4 ± 5.2	34.7 ± 4.8	38.2 ± 2.9	38.2 ± 4.5	<0.0001	t = -8.091
RBC count (10^5^ cells/µL)	4.2 ± 0.7	4.5 ± 0.5	4.7 ± 0.4	4.9 ± 0.6	<0.0001	t = -6.706
MCV (fL)	82.5 ± 3.99	85.0 ± 3.29	83.2 ± 1.85	84.1 ± 2.48	0.032	t = -2.165
MCH (pg)	26.2 ± 2.4	28.0 ± 1.9	28.4 ± 1.4	28.4 ± 2.1	<0.0001	t = -6.544
MCHC (g/dL)	31.2 ± 1.5	32.5 ± 0.77	32.2 ± 0.60	32.2 ± 0.77	<0.0001	t = -4.958
Platelet count (10^3^ cells/µL)	315.2 ± 55.3	292.8 ± 54.9	275.3 ± 46.4	276.7 ± 52.3	<0.0001	t = 4.491

In the pulmonary TB group (n = 76), disease severity at admission revealed minimal lesions in eight (10.5%), moderately advanced lesions in 38 (50.0%), and far-advanced lesions in 30 (39.5%) participants. At two and six months, minimal lesions were observed in six (10.9%) and five (11.4%), respectively, while moderately advanced cases decreased to 27 (49.1%) and 21 (47.7%). Cavitary lesions were present in 55 (72.4%) at admission, 43 (78.2%) at two months, and 33 (75.0%) at six months. Anemia was significantly prevalent in the TB group, with 44 (88%) participants at admission, dropping to 14 (25.5%) at two months and none at six months, compared to 12 (15.8%) in the control group (p < 0.0001). Anemia severity at admission included mild cases in 18 (36%), moderate in 20 (40%), and severe in six (12%) (Table 3).

**Table 3:** Classification and prevalence of disease severity and anemia types among pulmonary tuberculosis patients and control group TB: tuberculosis

Variables	Pulmonary TB group (n = 76) frequency (%)			Control group (n = 76) frequency (%)	p-value	Chi-square value (χ²)
	At admission (n = 76)	At 2 months (n = 55)	At 6 months (n = 44)			
Severity of disease						
Minimal lesion	8 (10.5%)	6 (10.9%)	5 (11.4%)	-	0.061	0.999
Moderately advanced	38 (50.0%)	27 (49.1%)	21 (47.7%)	-		
Far advanced	30 (39.5%)	22 (40.0%)	18 (40.9%)	-		
Cavitary lesions						
Yes	55 (72.4%)	43 (78.2%)	33 (75.0%)	-	0.750	0.573
No	21 (27.6%)	12 (21.8%)	11 (25.0%)	-		
Anemia	44 (88%)	14 (25.5%)	0 (0%)	12 (15.8%)	0.124	10.012
Microcytic anemia	12 (24%)	0 (0%)	0 (0%)	4 (5.3%)		
Normocytic anemia	30 (60%)	14 (25.5%)	0 (0%)	6 (7.9%)		
Macrocytic anemia	2 (4%)	0 (0%)	0 (0%)	2 (2.6%)		
Severity of anemia						
Mild	18 (36%)	10 (18.2%)	0 (0%)	7 (9.2%)	<0.0001	5.731
Moderate	20 (40%)	4 (7.3%)	0 (0%)	3 (3.9%)		
Severe	6 (12%)	0 (0%)	0 (0%)	2 (2.6%)		

At admission, serum ferritin levels were 95.2 ± 38.7 ng/mL, serum iron was 42.6 ± 15.3 µg/dL, and transferrin saturation was 13.9 ± 5.2% in the pulmonary TB group, significantly lower than in the control group (75.3 ± 28.1 ng/mL, 55.0 ± 14.2 µg/dL, and 17.5 ± 4.7%, respectively; all p < 0.0001). These parameters improved during treatment: at six months, serum ferritin decreased to 60.2 ± 22.4 ng/mL, serum iron increased to 70.8 ± 11.5 µg/dL, and transferrin saturation rose to 21.4 ± 3.9% (p < 0.0001). Inflammatory markers also showed improvement, with CRP levels decreasing from 35.6 ± 18.4 mg/L at admission to 8.0 ± 5.1 mg/L at six months (p < 0.0001) and ESR values dropping from 51.4 ± 23.0 mm/hr at admission to 13.1 ± 6.1 mm/hr at six months (p < 0.0001) (Table 4).

**Table 4:** Serum iron parameters and inflammatory markers in pulmonary tuberculosis patients and control group TB: tuberculosis; TIBC: total iron-binding capacity; ESR: erythrocyte sedimentation rate; CRP: C-reactive protein

Parameter	Pulmonary TB group (n = 76) mean ± SD			Control group (n = 76) mean ± SD	p-value	F-value
	At admission (n = 76)	At 2 months (n = 55)	At 6 months (n = 44)			
Serum ferritin (ng/mL)	95.2 ± 38.7	75.3 ± 28.1	60.2 ± 22.4	45.3 ± 22.1	<0.0001	F = 19.232
Serum iron (µg/dL)	42.6 ± 15.3	55.0 ± 14.2	70.8 ± 11.5	75.2 ± 18.5	<0.0001	F = 35.761
TIBC (µg/dL)	304.1 ± 44.2	316.6 ± 42.6	335.7 ± 38.4	344.5 ± 52.6	0.002	F = 4.253
Transferrin saturation (%)	13.9 ± 5.2	17.5 ± 4.7	21.4 ± 3.9	22.1 ± 6.8	<0.0001	F = 22.754
CRP (mg/L)	35.6 ± 18.4	15.2 ± 8.9	8.0 ± 5.1	5.6 ± 2.4	<0.0001	F = 45.198
ESR (mm/hr)	51.4 ± 23.0	29.3 ± 16.2	13.1 ± 6.1	12.0 ± 5.8	<0.0001	F = 39.701

Pearson correlation analysis showed a significant negative correlation between hemoglobin levels and TB severity (r = -0.58, p < 0.0001) and symptom duration (r = -0.49, p < 0.0001), highlighting the detrimental impact of advanced TB and prolonged illness. A positive correlation was observed between BMI within the normal range and hemoglobin levels (r = 0.45, p < 0.0001), as well as between lower CRP levels (<10 mg/L) and hemoglobin levels (r = 0.52, p < 0.0001) (Table 5).

**Table 5:** Correlation between hemoglobin levels and parameters in pulmonary TB group (n = 76) BMI: body mass index; TB: tuberculosis: CRP: C-reactive protein

Severity parameter	Correlation coefficient (r)	p-value
TB severity (far advanced)	-0.58	<0.0001
Duration of symptoms (>8 weeks)	-0.49	<0.0001
BMI (18.5-24.9 kg/m²)	0.45	<0.0001
CRP level (<10 mg/L)	0.52	<0.0001

## Discussion

The findings of this study highlight the significant hematological changes and high prevalence of anemia among patients with pulmonary TB. In our cohort, 44 out of 50 patients (88%) presented with anemia at admission, with normocytic anemia being the most prevalent type (60%). These results align with prior studies, such as de Mendonça et al., who reported anemia in 85% of TB patients, underscoring its status as a common and often overlooked complication of TB [15].

The high prevalence of anemia in TB patients is multifactorial, involving chronic inflammation, nutritional deficiencies, and the direct effects of *Mycobacterium tuberculosis *on hematopoietic function. Chronic inflammation elevates pro-inflammatory cytokines like IL-6, which stimulate hepcidin production. Hepcidin, a regulator of iron metabolism, inhibits iron absorption and mobilization, leading to ACD. Nutritional deficiencies, including iron, vitamin B12, and folate, exacerbated by the metabolic demands of TB, further impair erythropoiesis [7,8].

In India, where TB and malnutrition are highly prevalent, the combined effects of these conditions pose a significant public health challenge. Malnutrition not only predisposes individuals to TB but also worsens anemia by limiting nutrient availability. Addressing these issues requires a comprehensive approach integrating nutritional supplementation with TB treatment and anemia management [13].

The mean hemoglobin level in the pulmonary TB group was 10.10 ± 1.73 g/dL, significantly lower than the control group's 12.11 ± 1.57 g/dL (p < 0.0001). This reduction reflects the systemic impact of TB on erythropoiesis. A mean hemoglobin level of 10.10 g/dL falls into the category of moderate anemia per the WHO guidelines, potentially compromising oxygen delivery and exacerbating clinical symptoms like fatigue and breathlessness. These effects increase the disease burden and delay recovery [16,17].

Patients in this study were treated with a fixed-dose combination (FDC) regimen for ATT as per NTEP guidelines. While the FDC regimen simplifies treatment and improves adherence, gastrointestinal intolerance was reported in some cases, potentially affecting drug absorption and nutritional status. Addressing these adverse effects through dietary counseling and symptomatic management is critical to optimizing outcomes [12].

The clinical implications of anemia in TB patients are profound, as anemia exacerbates morbidity and hinders immune responses, potentially prolonging recovery and increasing the risk of poor treatment outcomes. These findings underscore the importance of routine anemia screening in TB patients and the implementation of integrated management strategies to address TB and its systemic complications [6].

Moreover, the correlation between TB severity and hemoglobin levels, with a coefficient of -0.58 (p < 0.0001), underscores the relationship between disease progression and hematological status. This finding is consistent with the results of Leon et al., and Chhabra et al., who demonstrated that severe TB is associated with lower hemoglobin levels and increased rates of anemia [18,19]. The negative correlation can be attributed to the inflammatory milieu created by TB, which includes elevated levels of cytokines such as IL-6 and TNF-α [20]. These cytokines inhibit erythropoiesis and promote iron sequestration, leading to anemia of chronic disease [21,22].

Our results also reveal a significant association between lower BMI and poorer hemoglobin status, with an average BMI of 18.9 ± 2.4 kg/m² in the pulmonary TB group, significantly lower than the control group's 22.1 ± 2.6 kg/m² (p < 0.0001). This observation supports the findings of Ko et al., who highlighted that malnutrition is prevalent in TB patients and exacerbates anemia [23]. The interplay between malnutrition and TB is complex; malnutrition not only compromises immune function, making individuals more susceptible to TB but also impairs hematopoiesis, resulting in a vicious cycle of worsening health outcomes [24].

Additionally, our study documented a significant decrease in serum ferritin levels over the treatment period, from 95.2 ± 38.7 ng/mL at admission to 45.3 ± 22.1 ng/mL at 6 months (p < 0.0001). While elevated ferritin levels are often observed in TB patients due to its role as an acute-phase reactant, the marked decline over six months suggests a dual effect: resolution of inflammation and possibly reduced iron stores due to dietary deficiencies or disease-related iron sequestration. In this cohort, the mean ferritin levels at baseline exceeded the upper threshold of normal (20-250 ng/mL in males and 10-120 ng/mL in females), reflecting the inflammatory response to TB rather than true iron sufficiency [25].

The clinical implications of declining ferritin levels are multifaceted. A reduction in ferritin may signal improved control of systemic inflammation, as indicated by a concurrent decrease in CRP levels from 35.6 ± 18.4 mg/L at admission to 5.6 ± 2.4 mg/L at six months (p < 0.0001). However, this decline must be carefully interpreted in the context of anemia. If ferritin levels drop below the lower limit of normal, this may indicate a transition to iron deficiency anemia, necessitating intervention. Excessive iron supplementation should be avoided in TB patients as it can potentially fuel bacterial growth and worsen clinical outcomes, underscoring the need for balanced nutritional management [26].

The observed correlation between decreasing CRP and ferritin levels aligns with findings from Landry et al. and Calderwood et al., who reported a similar resolution of systemic inflammation in TB patients undergoing treatment [27,28]. However, distinguishing between ferritin's role as an acute-phase reactant and true iron deficiency requires additional biomarkers, such as transferrin saturation or soluble transferrin receptor levels, which were not evaluated in this study.

Limitations

This study has several limitations that should be considered when interpreting the results. First, the sample size, although adequate for preliminary analysis, may limit the generalizability of our findings to larger populations. Second, the cross-sectional design restricts the ability to establish causal relationships between TB severity, anemia, and other variables. Third, potential biases in self-reported data regarding previous TB treatment and dietary habits may affect the accuracy of our findings. Additionally, the study did not account for all potential confounding factors, such as HIV co-infection, parasitic infestations, or other chronic conditions like diabetes and chronic kidney disease, which are prevalent in the Indian population and may impact anemia in TB. Lastly, the relatively short follow-up period may not capture long-term hematological outcomes and the full recovery potential of TB patients posttreatment.

Future research should focus on exploring cytokine profiles to elucidate the inflammatory mechanisms contributing to anemia, the role of micronutrient supplementation in improving hematological outcomes, and the long-term impact of anti-TB treatment on hematological recovery. Longitudinal studies with larger cohorts are essential to validate these findings and provide a deeper understanding of the interplay between TB, anemia, and systemic inflammation in endemic settings.

## Conclusions

In conclusion, our study highlights the complex interplay between pulmonary TB, anemia, and nutritional status, emphasizing the need for integrated management strategies that address both the infectious and hematological dimensions of the disease. The high prevalence of anemia in TB patients necessitates routine screening and targeted interventions, such as nutritional support and carefully managed iron therapy, to improve patient outcomes. However, the potential risks of iron therapy, including the promotion of bacterial growth, underline the importance of cautious and personalized treatment approaches.

Future research should prioritize exploring the mechanisms underlying anemia in TB, such as cytokine profiling, to better understand the inflammatory pathways involved. Additionally, therapies combining micronutrient supplementation and anti-inflammatory strategies could play a pivotal role in enhancing recovery. Such interventions, if effectively implemented, can significantly impact public health in high-burden countries like India, where TB and anemia coexist at alarming rates, potentially improving the quality of life and reducing the disease burden in affected populations.
